# Caffeine is a respiratory stimulant without effect on sleep in the short-term in late-preterm infants

**DOI:** 10.1038/s41390-021-01794-y

**Published:** 2021-10-30

**Authors:** Maija Seppä-Moilanen, Sture Andersson, Turkka Kirjavainen

**Affiliations:** New Children´s Hospital, and Pediatric Research Center, University of Helsinki and Helsinki University Hospital, Helsinki, Finland

## Abstract

**Background:**

Caffeine is widely used in preterm infants for apnea control. It has no effect on sleep in the only existing polysomnographic study including ten preterm infants Behavioral and polygraphic studies have conflicting results.

**Methods:**

We studied 21 late-preterm infants at a median gestational age of 36 weeks. Polysomnography was performed twice, at baseline on day 1 and on the day after the onset of caffeine treatment (20 mg/kg loading and 5 mg/kg morning maintenance dose).

**Results:**

Caffeine acted short term as a breathing stimulant with reduction of apneas, improved baseline SpO_2_ (*p* < 0.001), and decreased 95 percentile of end-tidal carbon dioxide level (*p* < 0.01). It also increased arousal frequency to SpO_2_ desaturations of more than 5% (*p* < 0.001). Caffeine did not affect sleep stage distribution, sleep efficiency, frequency of sleep stage transitions, appearance of REM periods, or the high number of spontaneous arousals. The median spontaneous arousal count was 18 per hour at baseline, and 16 per hour during caffeine treatment (*p* = 0.88).

**Conclusions:**

In late-preterm infants, caffeine has a clear short-term respiratory stimulant effect, and it increases the arousal frequency to hypoxia. However, caffeine does not appear to act as a central nervous system stimulant, and it has no acute effect on sleep quality.

**Impact:**

- Effects of caffeine on sleep in preterm infants has previously been investigated with only one full polysomnographic study including ten preterm infants. The study showed no effect.
- The current study shows that caffeine acts short term as a respiratory stimulant and increases arousal frequency to hypoxia.
- Although a potent central nervous system (CNS) stimulant in adults, caffeine does not seem to have similar acute CNS effect in late-preterm infants.
- The onset of caffeine treatment has no short-term effect on sleep stage distribution, sleep efficiency, frequency of sleep stage transitions, appearance of REM periods, or the high number of spontaneous arousals.

## Introduction

Caffeine is globally the most widely used psychoactive stimulant^1^. In addition to its central nervous system (CNS) stimulant effect, caffeine and other methyxanthines are respiratory stimulants. Due to its effects on breathing, caffeine is used in preterm infants for treatment and prevention of apneas^2,3^. In preterm infants, caffeine effectively decreases long central and mixed apneas and periodic breathing^3,4^. For infants born at less than 32–34 weeks of gestation, caffeine treatment is commonly started during the first day of life and discontinued at around 34–35 weeks of gestational age^3,5^. However, some preterm infants may continue to express apneas and periodic breathing, and could benefit from continuing treatment^4^. As used for prevention and treatment of apneas in preterm infants, caffeine improves long-term neurological outcome^6–9^. The mechanism of this positive effect is not properly confirmed, although the most readily available explanation is the reduction of apneas and repetitive hypoxia^3,4,10^. Another potential mechanism is direct neuroprotective action of caffeine through adenosine receptors^11–13^.

Sleep is important for the developing brain^14^. Disturbances in sleep distribution may cause neurological sequelae during this time of rapid brain maturation^15,16^. In animal studies, rapid eye-movement (REM) sleep deprivation has proven harmful^14,17^. In adults and adolescents, as a CNS stimulant, caffeine alters sleep quality by reducing total sleep time and sleep efficiency, prolonging sleep latency, and reducing subjective sleep quality^1^. In clinical practice, infants seem to sleep well even when on high doses of caffeine for apnea treatment. The effect of caffeine on sleep in preterm and term infants remains controversial and not widely studied^18–25^. The aim of this study was to investigate short-term effects of caffeine on sleep in late preterm infants with polysomnography (PSG).

## Methods

### Study design and patients

We performed PSG recordings in 21 infants born preterm in the neonatal units of Helsinki University Hospital, Helsinki, Finland. At the time of the study, the infants were clinically stable with no respiratory support or caffeine treatment. The studied infants were considered by the clinician in charge to need caffeine treatment for apneas with desaturations, or excessive periodic breathing. The study infants underwent full PSG studies to investigate respiratory events and sleep. On day 1, a baseline recording was performed followed by administration of a caffeine citrate loading dose of 20 mg/kg. Caffeine treatment was continued with a daily dose of 5 mg/kg. On day 2, after onset of caffeine treatment, a second recording was performed.

The Helsinki University ethics committee approved the study. Parents provided written consent forms and did not receive any monetary compensation for participation.

### Polysomnography

The PSG setup followed the recommendations of the American Academy of Sleep Medicine (AASM)^26^. It comprised monitoring of electroencephalography (EEG) channels (C4-M1, Cz-Fz, Cz-O2, and O2-M1), left and right electro-oculography (EOG) channels, nasal airflow (pressure sensor), respiratory movements (abdominal band), chin and diaphragm electromyography (EMG), electrocardiography (ECG, lead II position), pulse oximeter oxygen saturation (SpO_2_) with a 4-s averaging interval, and end-tidal carbon dioxide (EtCO_2_). The PSG recordings were done using Siesta PSG equipment (Compumedics, Abbotsford, Australia).

The PSG recordings were converted into European Data Format (EDF) and transferred into Embla® RemLogic™ PSG software (Natus Medical Inc., Pleasanton, CA) for both visual (T.K.) and automatic scoring analysis. The completion of data analysis was done by an extensive research special purpose software.

The sleep stage analysis was performed visually, recognizing wakefulness, non-REM (NREM) sleep, and REM sleep. An arousal was defined as a period of 3 s or more with a sustained increase in chin EMG with or without changes in the EEG signals. Heart rate was not used as an indicator. Central, obstructive, and mixed apneas, and periodic breathing were recognized. We determined respiratory pauses of 4 s or more as apneas. Apnea and periodic breathing definitions, as well as arousal definitions used in this study are presented in Table 1 and have also been previously described in detail^4,27^. We scored breathing effort visually from the PSG analysis based on the diaphragm EMG on a scale of none (0) to maximal (2) effort in apneas with obstructive breaths. Heart rate variability (HRV) was measured from periods of deep NREM sleep.

**Table 1:** Apnea and arousal definitions used in the current study.

Apnea	Definition	Duration
Central apnea	Apnea with no breathing movements	Pause in breathing lasting: >2 breathing cycles and ≥4 s
Obstructive apnea	Apneas with breathing movements but no airflow	All obstructive pauses of breathing
Mixed apnea	Apneas commencing as central but showed obstructive respiratory movements	All mixed apneas
Apnea of prematurity	Apnea with: Heart rate decrease to <100 beats per minute, or drop in SpO_2_ to <80%, or apnea length of >20 s	
**Arousal**	**Definition**	**Duration**
Arousal	A sustained increase in chin and diaphragm EMG from baseline recording excluding sucking of the dummy, or gross body movements causing artefacts in ECG, EEG, respiratory signal	≥3 s
Apnea arousal	An arousal appearing during an apnea, or within 5 s after the end of an apnea	≥3 s
Long arousal or awakening	Same as arousal	≥15 s

*EMG* electromyogram, *ECG* electrocardiogram, *EEG* electroencephalogram.

### Statistical methods and analysis

We used the non-parametric Wilcoxon signed-rank test for pairwise comparison as the number of study infants was limited to 21 and the majority of the dependent variables were not normally distributed. Results were noted significant at *p* < 0.05. For the statistical analysis we used SPSS® Statistics software versions 27 (IBM, Armonk, NY).

## Results

At the time of the study, the median age of the 21 infants was 4.7 (interquartile range, IQR 2.8–7.1) weeks, and gestational age 36 (IQR 35–36) weeks. They were born at a median of 31 (IQR 28 to 33) weeks of gestation with a median birth weight of 1.610 (IQR 1.140–2.190) kg. None of the infants received respiratory support or supplemental oxygen directly before the study. Sixty-seven percent of the infants had previous caffeine treatment, which was discontinued at a median of 8 days before the study onset. The demographic data are presented in more detail in Table 2.

**Table 2:** Demographic data of the study infants.

Infants (*n*)	21
Female	13 (62%)
Gestational age at birth (weeks)	31.1 (28.4–33.6)
Weight at birth (kg)	1.610 (1.140–2.190)
BPD	5 (24%)
GMV-IVH	3 (14%)
Grade 1 to 2	2 (9.5%)
Grade 4	1 (4.8%)
Age at study (weeks)	4.7 (2.8–7.1)
Gestational age at study (weeks)	35.7 (35.0–36.3)
Weight at study (kg)	2.240 (2.015–2.595)
Infants with caffeine previously	14 (67%)
Caffeine-free period before study (days)	8 (7–11)
Gestational age (weeks) at cessation of previous caffeine treatment	34.0 (33.7–35.0)

*BPD* bronchopulmonary dysplasia, *GMH-IVH* germinal matrix or intraventricular hemorrhage. Results presented as median (interquartile range) or number (percentage).

### Effect of caffeine on breathing

**Fig. 1:**
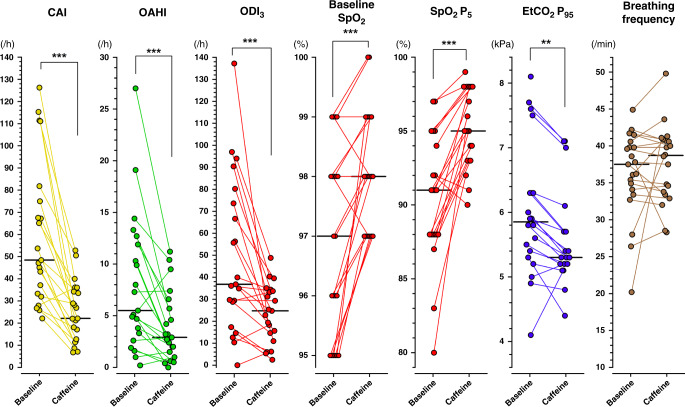
Apnea and respiratory results presented as individual changes. Caffeine acted as a ventilatory stimulant in late-preterm infants. Caffeine treatment decreased the central apnea index (CAI), the obstructive apnea-hypopnea index (OAHI), and oxygen desaturation of over 3% from baseline (ODI_3_). Baseline median pulse oximeter oxygen saturation (SpO_2_) and the 5th percentile SpO_2_ level increased with caffeine treatment. Breathing frequency remained unchanged, but the end-tidal carbon dioxide (EtCO_2_P95) level decreased with caffeine. See also Supplementary Table S1 for more specific data on EtCO_2_ and breathing frequency values. /h per hour of sleep, /min per minute, kPa kilopascal, **P < 0.01, ***P < 0.001.

Caffeine acted as a short-term breathing stimulant (Fig. 1). Caffeine reduced the number of apneas (*p* < 0.001), frequency of oxygen desaturation (*p* < 0.001), increased median SpO_2_ levels (*p* < 0.001), and decreased the high 95th percentile EtCO_2_ level (*p* < 0.01), but caffeine did not show significant effect on breathing frequency (Supplementary Table S1).

### Sleep characteristics and spontaneous arousals

**Fig. 2:**
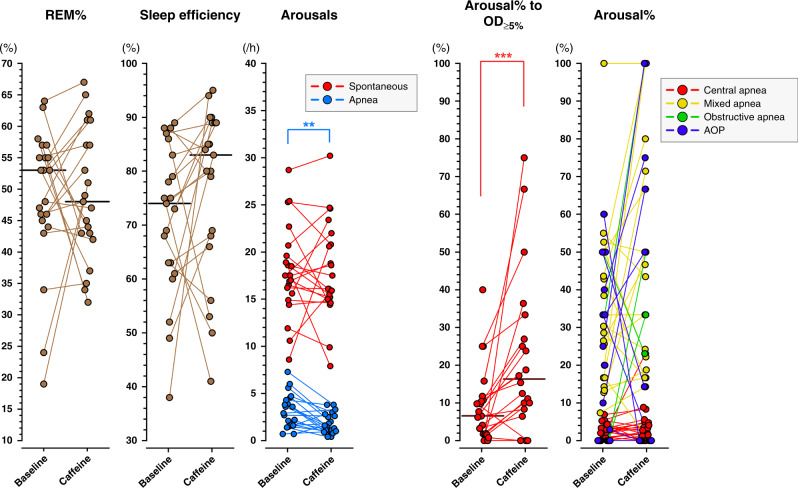
Sleep and arousal results presented as individual changes. Sleep parameters remained unchanged with caffeine treatment, and caffeine did not act as a central nervous system stimulant in late-preterm infants. The amount of rapid-eye-movement (REM) sleep, sleep efficiency, and the frequency of spontaneous arousals did not change with caffeine treatment. Apnea arousals decreased with caffeine treatment due to the reduction in apneas. Caffeine treatment increased the rate of arousal to desaturation of a minimum 5% units (OD_≥5%_), but it had no effect on arousal to apneas. See also Table 3 for more specific data on sleep parameters, Table 4 for arousal percentages also in varying sleep stages, Table 5 for arousal to desaturation, and supplementary Table S2 for data on arousal to varying types of apneas. /h per hour of sleep, AOP apnea of prematurity, **P < 0.01, ***P < 0.001.

Sleep characteristics and arousal data are presented in Tables 3 and 4 and Fig. 2. Baseline PSG recordings lasted longer than recordings after the onset of caffeine treatment (*p* = 0.002) but there was no significant difference in sleep efficiency (*p* = 0.16). All the main sleep attributes remained similar in both study phases (Table 3); sleep stage distribution, frequency of sleep stage transitions, REM sleep latency, and other characteristics of REM sleep showed no significant changes during caffeine treatment. Spontaneous arousals were frequent and more common in REM than in NREM sleep both during baseline PSG recording and after the onset of caffeine treatment (Table 4). Figure 2 shows individual changes in sleep and arousal parameters.

**Table 3:** Polysomnographic recording times and sleep characteristics.

	1. Baseline	2. Caffeine	*P* value
Recording time (minutes)	223 (188–284)	183 (165–199)	0.002
Sleep efficiency (%)	74.0 (61.5–84.5)	83.0 (67.0–89.0)	0.16
TST (minutes)	166 (135–200)	152 (120–164)	0.054
REM of TST (%)	53.0 (44.0–56.0)	48.0 (42.5–59.0)	0.63
NREM of TST (%)	47.0 (44.0–55.5)	52.0 (41.0–57.0)	0.64
REM latency	1.0 (0.0–20.8)	0.0 (0.0–11.0)	0.55
Sleep stage transitions per hour	21.9 (18.45–27.6)	24.50 (18.9–28.7)	0.65
REM periods (minutes)			
Shortest	4.5 (4.0–6.0)	4.5 (4.0–6.3)	0.97
Longest	25.5. (16.0–38.8)	23.0 (15.5–37.3)	0.95
Average time	11.0 (8.3–15.2)	12.5 (9.0–16.2)	0.38
Average interval	21.4 (13.3–29.7)	17.4 (14.1–27.1)	0.39

Main sleep parameters remained unchanged during caffeine treatment. *TST* total sleep time, *REM* rapid eye-movement sleep, *NREM* non-rapid eye-movement sleep. Results presented as median (interquartile range).

*P* = significance according to Wilcoxon signed-rank test of two related samples

**Table 4:** Number of arousals from sleep.

	1. Baseline	2. Caffeine	*P* value
Total arousal count per hour			
TST	20.5 (18.6–24.5)	19.3 (16.4–23.1)	0.20
NREM	14.0 (9.1–17.0)	11.6 (8.8–14.9)	0.51
REM	29.1 (23.0–35.2)	26.2 (22.6–29.1)	0.048
* P* NREM vs. REM	<0.001	<0.001	
Spontaneous arousal count per hour			
TST	17.5 (15.3–20.2)	16.1 (14.9–21.4)	0.88
NREM	11.8 (7.0–14.2)	11.3 (8.5–14.5)	0.31
REM	26.5 (19.1–29.3)	23.2 (20.0–26.2)	0.24
* P* NREM vs. REM	<0.001	<0.001	
Apnea arousal count per hour			
TST	2.9 (1.6–4.5)	1.5 (1.0–2.7)	0.002
NREM	2.2 (1.3–4.2)	0.9 (0.0–1.3)	<0.001
REM	4.4 (2.4–6.4)	2.1 (1.3–4.7)	0.012
* P* NREM vs. REM	0.005	0.001	

*TST* total sleep time, *NREM* non-rapid eye-movement sleep, *REM* rapid eye-movement sleep, *AOP* apnea of prematurity.

Results presented as median (interquartile range).

*P* = significance according to Wilcoxon signed-rank test of two related samples.

The rate of spontaneous arousals from sleep did not differ with caffeine treatment. Apnea arousals were less frequent during caffeine treatment due to the decrease of apneas. Arousals were more common in REM than in NREM sleep.

### Arousal to apneas and hypoxia

Periodic breathing, and central, mixed, and apnea of prematurity defined apneas decreased with caffeine (Fig. 1), as we have previously shown^4^. Arousal responses to apneas were low during both baseline and caffeine (Fig. 2). The median percentage of all apneas that led to arousal was 5% at baseline and 8% during caffeine treatment (*p* = 0.10). Caffeine treatment did not affect the frequency of arousal responses to different types on apnea (Fig. 2 and Supplementary Table S2). Similar to the frequency of spontaneous arousals, apnea arousals were more common in REM sleep than in NREM sleep (*p* < 0.01) (Table 4).

Desaturation was not a potent factor in inducing arousals in these preterm infants. However, caffeine treatment increased the arousal percentage to desaturations of over 5% units (Fig. 2, Arousal% to OD_≥5%_), especially in REM sleep (*p* < 0.001). Also, arousal to desaturations to SpO_2_ of less than 90% was increased in REM sleep (*p* = 0.03), and to less than 95% both in REM and NREM sleep (*p* < 0.001 and *p* = 0.01, respectively) (Table 5).

**Table 5:** Percentage of desaturations leading to arousal.

	1. Baseline	2. Caffeine	*P* value
Arousal to desaturations to <90%			
TST	3.6 (0.0–13.4)	14.8 (0.0–32.1)	0.011
NREM	0.6 (0.0–8.2)	12.1 (0.0–44.6)	0.20
REM	4.4 (0.0–16.7)	20.0 (0.0–36.4)	0.028
*P* NREM vs. REM	0.75	0.17	
Arousal to desaturations to <95%			
TST	5.3 (1.8–8.5)	14.9 (7.3–31.1)	<0.001
NREM	1.3 (0.0–5.4)	12.1 (0.0–44.6)	0.011
REM	8.0 (5.1–12.2)	16.5 (11.1–30.4)	<0.001
*P* NREM vs. REM	0.007	1.0	
Arousal to desaturations of ≥5%			
TST	6.6 (1.7–11.3)	16.3 (8.8–31.7)	<0.001
NREM	0.9 (0.0–6.2)	3.3 (0.0–33.3)	0.11
REM	10.6 (4.1–16.3)	24.0 (16.8–46.6)	<0.001
*P* NREM vs. REM	0.059	0.19	

*TST* total sleep time, *NREM* non-rapid eye-movement sleep, *REM* rapid eye-movement sleep.

Results presented as median (interquartile range).

*P* = significance according to Wilcoxon signed-rank test of two related samples

Caffeine increased the arousal rate to desaturations of SpO_2_ to less than 90% and 95%, and to desaturation of a minimum 5% units from baseline SpO_2_ level.

### Heart rate variability

Low-frequency variability (LFV), high-frequency variability (HFV), and total power (TP) showed no differences from baseline to caffeine treatment (Supplementary Table S3).

## Discussion

Our study shows that although caffeine acts as a respiratory stimulant in late-preterm infants, it does not seem to have a clear short-term CNS stimulant effect. Caffeine reduced the number of apneas, increased the frequency of arousals to hypoxia, improved SpO_2_ baseline, and decreased the 95th percentile EtCO_2_ level. The effect on hypoxia-related arousals was more pronounced in REM than in NREM sleep. Caffeine treatment did not have a clear short-term effect on sleep. After the onset of caffeine treatment, sleep stage distribution, sleep efficiency, frequency of sleep stage transitions, appearance of REM periods, and the high number of spontaneous arousals remained unaffected. We suggest that the increase in arousal tendency to hypoxia is due to an increase in hypoxic ventilatory drive rather than an increased general arousability.

### The effect of caffeine on sleep

It is generally assumed that after 27–28 weeks of gestational age premature infants start to exhibit defined sleep states or stages^17,28,29^. REM sleep is clearly established in infants older than 30 weeks of gestation^30^. Sleep is an important state in the development of infants. In animal studies, especially the development of REM sleep is necessary for normal brain development. Deprivation of REM sleep in newborn rats causes, for example, anxiety and disturbed sleep, and a lack of brain plasticity in adulthood^14^.

Although caffeine has a clear effect on sleep quality in adults^1^, the impact on sleep in preterm infants is not as evident. There are some studies concentrating on this topic showing contradictory results^18–25^. An observational study by Thoman et al.^18^ implied that preterm infants previously treated with theophylline spend less time in active sleep than controls or full-term infants. Similar findings with caffeine were noted by Koch et al.^23^ during caffeine treatment with an observational study setup. They found caffeine to increase wakefulness and alertness by decreasing active sleep while the amount of quiet sleep remained unchanged during the first five days of caffeine treatment. In contrast, a polygraphic study by Dietrich et al.^19^ in nine preterm apneic infants showed just the opposite finding shortly after acute caffeine administration. Hayes et al.^24^ demonstrated in a video and polygraphic setting that preterm infants who had been treated with methyxanthines for more than 5 days had lower arousal rates and shorter duration of sleep-related spontaneous movements than controls.

A PSG based study of Curzi-Dascalova et al.^20^ performed during maintenance caffeine treatment had findings similar to our short-term results showing that caffeine treatment does not have a clear effect on sleep. A loading dose of caffeine was given a minimum 3 days in advance. The findings of Curzi-Dascalova et al. are supported by other small studies both with investigations of chronic and short-term methylxanthine treatment^20–22^. The CAP (caffeine for apnea of prematurity) trial showed with neurocognitive testing, PSG, actigraphy, and sleep questionnaires that caffeine treatment for apnea of prematurity (AOP) and AOP prevention has positive long-term neurocognitive action without long-term effects on sleep at ages 5–12 years^7–9,31^.

Most of the few studies of the effects of methylxanthines on sleep in preterm infants have been conducted during maintenance methylxanthine treatment^20,21,23–25^. Only a few have investigated short-term effects of caffeine as in the current study^19,22,23^. Koch et al.^23^ studied both short-term and maintenance treatment of five days. At the beginning of caffeine treatment, due to slow elimination in (preterm) infants, caffeine levels accumulate. After 5–7 days, caffeine concentration starts to slowly decrease due to an increase in caffeine clearance^32^.

### The effect of caffeine on arousal to apneas and desaturation

We show that caffeine significantly reduces the number of apneas and hypoxic episodes. Arousal responses to apneas remain unaffected but caffeine increased the frequency of arousals to hypoxic events. Thoppil et al.^25^ found similar results with maintenance theophylline treatment. However, in the current study, even during caffeine treatment the rate of arousal to desaturation of a minimum 5% (units) from baseline SpO_2_ was only 24% in REM sleep and 3% in NREM sleep. Our results concur with previous studies showing hypoxia often to fail in causing arousals in infants, especially in NREM sleep^27,33,34^. As the number of spontaneous arousals remained unaffected by caffeine, we suggest the increased arousal frequency to hypoxia to be due to an increase in hypoxic ventilatory drive instead of an increase in general arousability.

### Caffeine mechanism of action

Caffeine is a CNS and respiratory stimulant^1^. Both actions are supposedly mediated though antagonizing adenosine receptors A_1_ and A_2A_^1,35^. In adults and adolescents, caffeine alters sleep quality by reducing total sleep time, reducing sleep efficiency, prolonging sleep latency, and reducing subjective sleep quality^1^. Preterm and term infants are commonly exposed to caffeine during fetal life through the placenta, and after birth through breast milk. Caffeine treatment is currently a part of common practice for prevention and treatment of apneas in preterm infants born at less than 34 weeks of gestation^5^. As treatment for apneas or their prevention, caffeine doses are at least 10-folds greater than through exposure from breast milk. With these high dosages, caffeine likely affects adenosine A_3_ receptors and GABA receptors in addition to A_1_ and A_2A_ receptors in the CNS^36^.

Caffeine is metabolized and excreted differently in preterm and term infants than in older children and adults. Preterm infants excrete most caffeine through the kidneys without significant liver metabolism. The hepatic metabolism takes over the direct excretion by 6–8 months of life. Because unmetabolized caffeine is excreted slowly, the half-life of caffeine in preterm and term infants ranges from 40–230 h, decreasing to adult levels of 2–5 h during the first year of life^37,38^. We find it unlikely that delayed excretion and a longer half-life would explain the differences in action of caffeine between preterm infants and adults. Yet, the differences in direct CNS mechanism of action seems evident.

### Limitations of the study

Sleep stages in term and preterm infants may be hard to differentiate. Accurate evaluation of different sleep stages generally requires EEG, eye movement (with EOG), and chin EMG confirmation^39^. With preterm infants, sleep stage definition is more complex. The EEG of preterm infants is immature. However, even among preterm infants, sleep stages may be assessed by respiration, heart-beat parameters, movements, and EMG tone^26,29,40^.

A consensus for arousal definition in preterm infants is lacking. Therefore we have in part applied the criteria of The International Pediatric Workgroup on Arousals for infants aged 1–6 months^27,41^.

Long-term follow-up in preterm infants previously treated with caffeine showed no effect on sleep and assures the safety of caffeine treatment in these infants^31^. We studied only short-term effects of caffeine and suggest caution when extrapolating these effects for long-term use. However, it is likely that the effects remain similar.

## Conclusions

Caffeine has a clear respiratory stimulant effect in late-preterm infants but it does not appear to act as a CNS stimulant. A high loading dose of caffeine 20 mg/kg does not affect sleep stage distribution, sleep efficiency, frequency of sleep stage transitions, appearance of REM sleep periods, or the high number of spontaneous arousals. Caffeine increases the arousal frequency to SpO_2_ desaturations. This is suggested to be caused by an increase in hypoxic ventilatory drive.

## Supplementary information

Supplementary information
