# Correction to: Caffeine is a respiratory stimulant without effect on sleep in the short-term in late-preterm infants

**DOI:** 10.1038/s41390-022-02092-x

**Published:** 2022-05-16

**Authors:** Maija Seppä-Moilanen, Sture Andersson, Turkka Kirjavainen

**Affiliations:** New Children´s Hospital, and Pediatric Research Center, University of Helsinki and Helsinki University Hospital, Helsinki, Finland

Correction to: *Pediatric Research* 10.1038/s41390-021-01794-y, published online 30 October 2021

In the original version of this article, there was an incorrect information in the legends explaining *P* value (**P) of figures 1 and 2 respectively. The legends should read as follows. The original article has been corrected.

Fig. 1 **Apnea and respiratory results presented as individual changes**. Caffeine acted as a ventilatory stimulant in late-preterm infants. Caffeine treatment decreased the central apnea index (CAI), the obstructive apnea-hypopnea index (OAHI), and oxygen desaturation of over 3% from baseline (ODI_3_). Baseline median pulse oximeter oxygen saturation (SpO_2_) and the 5th percentile SpO_2_ level increased with caffeine treatment. Breathing frequency remained unchanged, but the end-tidal carbon dioxide (EtCO_2_P95) level decreased with caffeine. See also Supplementary Table S1 for more specific data on EtCO_2_ and breathing frequency values. /h per hour of sleep, /min per minute, kPa kilopascal, **P < 0.01, ***P < 0.001.

Fig. 2 **Sleep and arousal results presented as individual changes**. Sleep parameters remained unchanged with caffeine treatment, and caffeine did not act as a central nervous system stimulant in late-preterm infants. The amount of rapid-eye-movement (REM) sleep, sleep efficiency, and the frequency of spontaneous arousals did not change with caffeine treatment. Apnea arousals decreased with caffeine treatment due to the reduction in apneas. Caffeine treatment increased the rate of arousal to desaturation of a minimum 5% units (OD_≥5%_), but it had no effect on arousal to apneas. See also Table 3 for more specific data on sleep parameters, Table 4 for arousal percentages also in varying sleep stages, Table 5 for arousal to desaturation, and supplementary Table S2 for data on arousal to varying types of apneas. /h per hour of sleep, AOP apnea of prematurity, **P < 0.01, ***P < 0.001.

