# Supplementary material for: Caffeine is a respiratory stimulant without effect on sleep in the short-term in late-preterm infants
Source: Pediatr Res. 2021 Oct 30;92(3):776–82. doi: 10.1038/s41390-021-01794-y (PMC9556325; doi:10.1038/s41390-021-01794-y)
Supplement: Supplementary file 1 — Supplementary information [file 41390_2021_1794_MOESM1_ESM.docx]

**Caffeine is a respiratory stimulant without effect on sleep in late-preterm infants**

Maija Seppä-Moilanen^1^, Sture Andersson^1^, Turkka Kirjavainen^1^

^1^Children´s Hospital, and Pediatric Research Center, University of Helsinki and Helsinki University Hospital, Helsinki, Finland

Supplementary data

Supplementary table S1: End-tidal carbon dioxide and breathing frequency data

Supplementary table S2: Percentage of apneas that led to arousals

Supplementary table S3: Heart rate variability data

| **Table S1─** End-tidal carbon dioxide and breathing frequency data | | | |  |  |
| --- | --- | --- | --- | --- | --- |
|  | **1. Baseline** | **2. Caffeine** | ***P*** | | |
| **EtCO_2_** |  |  |  | | |
| **TST** | 5.6 (5.3 to 6.3) | 5.3 (5.2 to 5.7) | 0.004 | | |
| **NREM** | 5.9 (5.3 to 6.4) | 5.4 (5.1 to 5.9) | 0.003 | | |
| **REM** | 5.8 (5.4 to 6.3) | 5.4 (5.1 to 6.0) | 0.008 | | |
| ***P* (NREM vs. REM)** | 0.18 | 0.94 |  | | |
| **Breathing frequency** |  |  |  | | |
| **TST** | 37.5 (33.7 to 40.2) | 38.7 (33.5 to 40.9) | 0.78 | | |
| EtCO_2_= end-tidal carbon dioxide, TST=total sleep time, NREM=non-rapid eye-movement sleep, REM=rapid eye-movement sleep.  Results presented as median (interquartile range).  *P*= significance according to Wilcoxon signed rank test of two related samples | | | | |  |

| **Table S2─** Percentage of apneas that led to arousals | | | | |  |
| --- | --- | --- | --- | --- | --- |
|  | **1. Baseline** | **2. Caffeine** | ***P*** | | |
| **All apneas** | 4.8 (2.6 to 8.4) | 7.9 (3.4 to 9.6) | 0.10 | | |
| **Central apneas** | 2.2 (0.85 to 3.8) | 2.7 (1.5 to 4.8) | 0.17 | | |
| **Obstructive apneas** | 0.0 (0.0 to 50.0) | 0.0 (0.0 to 33.3) | 0.14 | | |
| **Mixed apneas** | 30.3 (16.7 to 50.0) | 38.4 (17.2 to 70.2) | 0.19 | | |
| **AOP defined apneas** | 20.0 (0.0 to 40.0) | 7.1 (0.0 to 72.9) | 0.78 | | |
|  |  |  |  | | |
|  |  |  | |  |  |
| AOP=apnea of prematurity.  Results presented as median (interquartile range).  *P*= significance according to Wilcoxon signed rank test of two related samples | | | | |  |

| **Table S3─** Heart rate variability data | | | | |  |
| --- | --- | --- | --- | --- | --- |
|  | **1. Baseline** | **2. Caffeine** | ***P*** | | |
| **LFV** | 3.5 (3.2 to 3.7) | 3.5 (3.3 to 3.6) | 0.97 | | |
| **HFV** | 2.6 (2.5 to 3.0) | 2.8 (2.5 to 2.9) | 0.47 | | |
| **TP** | 3.8 (3.5 to 3.9) | 3.8 (3.6 to 4.0) | 0.75 | | |
| **LF/HF ratio** | 1.3 (1.2 to 1.4) | 1.3 (1.2 to 1.4) | 0.19 | | |
|  |  |  |  |  |  |
|  |  |  | |  |  |
| Results presented as median (interquartile range).  *P*= significance according to Wilcoxon signed rank test of two related samples | | | | |  |
